# Low Rate of Urological Complications in Pediatric Kidney Transplantation Using a Stentless Ureteral Anastomosis

**DOI:** 10.1111/petr.70431

**Published:** 2026-08-13

**Authors:** Gaetano Ciancio, Jeffrey J. Gaynor, Mahmoud Morsi, Henry Ocando Nava, Matthew Gaynor, Marissa Defreitas, Jayanthi Chandar

**Affiliations:** ^1^ Department of Surgery Jackson Memorial Hospital, University of Miami Miller School of Medicine Miami Florida USA; ^2^ Department of Urology Jackson Memorial Hospital, University of Miami Miller School of Medicine Miami Florida USA; ^3^ Miami Transplant Institute, Jackson Memorial Hospital, University of Miami Miller School of Medicine Miami Florida USA; ^4^ Division of Pediatric Nephrology, Department of Pediatrics Jackson Memorial Hospital, University of Miami Miller School of Medicine Miami Florida USA

**Keywords:** stenosis, stentless, urinary leak

## Abstract

**Background:**

Pediatric kidney transplant recipients are susceptible to a range of complications that may compromise allograft function and are associated with significant morbidity. Urologic complications represent an important subset that may necessitate multiple radiological and/or surgical interventions.

**Methods:**

Our previously reported modified extravesical ureteroneocystostomy technique was designed to avoid routine stent placement at the time of transplant as well as to reduce postoperative urologic complications. Here, we reviewed 107 consecutive pediatric kidney transplant recipients transplanted at our center since 2014 who received no stent placement at the time of transplant.

**Results:**

The observed percentage who developed a urologic complication during the first 12 months post‐transplant was 3.7% (4/107), and only 2.8% (3/107) required surgical intervention. Ureteral obstruction/stricture was observed in 2.8% (3/107), with two being due to UPJ obstruction, and one being due to the occurrence of multiple kidney stones that occluded the distal ureter/ureterovesical junction (UVJ). No urinary leaks, 0.0% (0/107), were observed during the first 12 months post‐transplant. Additionally, poor bladder compliance along with Grade 4 vesicoureteral reflux occurred in one patient. One patient (who developed a UPJ obstruction) was successfully managed with interventional radiology alone. The other three patients required surgical intervention: ureteral reimplantation for the patient having UVJ obstruction due to multiple kidney stones, pyeloureterostomy in one patient with a UPJ obstruction, and bladder augmentation along with (ureter, ileum, and ileal) Monti Mitrofanoff in the patient having poor bladder compliance.

**Conclusions:**

In pediatric kidney transplantation, our extravesical ureteroneocystostomy technique without routine ureteral stent placement was associated with a low incidence of post‐transplant urological complications.

## Graphical abstract

Among 107 consecutive pediatric kidney transplant recipients who received no stent placement at the time of transplant, the observed percentage who developed a urologic complication during the first 12 months post‐transplant was 3.7% (4/107). Our experience demonstrates that a standardized stentless extravesical ureteroneocystostomy technique in pediatric kidney transplantation can be performed safely with a low incidence of early post‐transplant urologic complications.

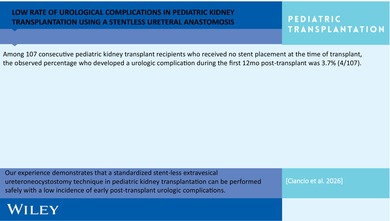

## 1 Introduction

Despite advances in surgical techniques in kidney transplantation, urologic complications such as ureterovesical junction (UVJ) stenosis or stricture, ureteral necrosis, and urinary leak may still occur in the early post‐transplant period following pediatric kidney transplantation with an incidence range of 3%–34% [1, 2]. These complications are associated with various potentially unfavorable outcomes, including morbidity, repeat hospitalizations, graft failure, and even mortality [3]. Some urologic complications may require multiple radiological and/or surgical interventions to achieve complete resolution [4].

The transplanted ureter is the principal source of most post‐transplant urologic complications. To mitigate this risk, routine placement of a ureteral stent at the time of transplant has been advocated. However, its universal use remains controversial in both adult [5, 6] and pediatric kidney transplant recipients [7], although the overall results reported in two meta‐analyses of mainly adult kidney transplant recipients support routine stent placement at the time of transplant [5, 6].

Our transplant program has implemented a standardized, stentless ureteroneocystostomy technique specifically developed to reduce the incidence of post‐transplant urologic complications in both children and adults. This approach is designed to promote secure healing of the ureterovesical anastomosis while minimizing or potentially eliminating the need for routine prophylactic ureteral stent placement at the time of transplant [8].

In this report, we detail the incidence and spectrum of urological complications in 107 consecutive pediatric kidney‐alone transplant recipients who underwent transplantation at our center since 2014 using our standardized ureteral anastomosis technique (performed by 2 of the transplant surgeons at our center), without the routine use of prophylactic ureteral stent placement.

## 2 Materials and Methods

### 2.1 Data Description

Between January 13, 2015, and April 10, 2025, a total 107 consecutive pediatric (age < 19 years) kidney‐alone transplant recipients at the Miami Transplant Institute underwent a novel extravesical ureteroneocystostomy performed without ureteral stenting (performed by 2 of the transplant surgeons at our center). This study is in accordance with the University of Miami Institutional Review Board and Helsinki Declaration (as revised in 2013), and all patients (or their guardians) signed an informed consent prior to transplant.

It should be noted that this study was not designed by retrospectively selecting only patients without ureteral stents. Rather, it reflects the routine surgical practice of the senior transplant surgeon (G.C.), who performed the vast majority of the pediatric kidney transplants at our center since 2014. His standard technique is ureteroneocystostomy without routine ureteral stent placement at the time of transplant. Also included were the cases of one of the other transplant surgeons (M.M.), who uses the same surgical techniques and without performing routine ureteral stent placement at the time of transplant. Therefore, the study represents a consecutive cohort managed with a consistent surgical approach rather than a selected subgroup.

Also of note, patients who underwent a more conventional extravesical ureteroneocystostomy (performed by one of the other transplant surgeons at our center) were not included here. During the study period, the great majority of pediatric kidney transplants were performed by two surgeons (G.C. and M.M.); thus, the number of pediatric transplants performed with alternative techniques by other surgeons was quite small (although more common among adult cases).

In terms of pretransplant urological evaluation, all pediatric kidney transplant candidates underwent a renal and bladder ultrasound screening. Patients with congenital anomalies of the kidney and urinary tract, abnormal ultrasound findings, lower urinary tract dysfunction, recurrent urinary tract infections (UTIs), and/or other suspected urological abnormalities were evaluated by the pediatric urology service. Additional diagnostic studies and any required interventions were performed at the discretion of the pediatric urologist; urological clearance was always obtained before transplantation.

All transplanted deceased donor (DD) kidneys had been connected to the LifePort Renal Preservation Machine prior to transplant using kidney perfusion solution (KPS‐1).

In performing the surgical technique for pediatric transplant recipients, the allograft was brought into the surgical field and detailed steps of the surgical approach, including the transplant technique and ureteral anastomosis have been described in previous publications [8, 9]. Regarding the ureteral anastomosis, Ciancio et al. [8] highlighted the advantages in using a previously unreported extravesical ureteroneocystostomy technique without ureteral stenting, that is, mobilized bladder, longer spatulation of the ureter, inclusion of bladder mucosa with detrusor muscle layer in the ureteral anastomosis, and use of a right‐angle clamp in the ureteral orifice during suturing in the attempt to ensure it does not become stenosed.

Clinical outcomes that occurred during the first 12 months post‐transplant were analyzed. Since the date of last follow‐up was April 30, 2026, all patients were followed for at least 12 months post‐transplant.

All patients in this study had a routine ultrasound of the kidney allograft performed during the first week post‐transplant. All urologic complications that developed during the first 12 months post‐transplant were recorded. Suspected urologic complications were evaluated with renal ultrasonography, MAG‐3 (mercaptoacetyltriglycine) scan, and/or antegrade nephrostogram. Urologic complications were treated by either percutaneous radiological procedures, surgery, or a combination of both. Suspected ureteral stenosis was evaluated along with serial monitoring for BK virus replication (in urine and blood). Voiding cystourethrogram was not routinely performed, as routine evaluation for vesicoureteral reflux (VUR) was not considered to be necessary in our transplant recipients; therefore, only symptomatic reflux was evaluated and considered as a urologic complication.

### 2.2 Statistical Analysis

The primary outcome variable for this study was the occurrence of a urologic complication (urinary leak or ureteral obstruction/stricture) during the first 12 months post‐transplant. Secondary outcome variables included the (separate) occurrence of a urinary leak during the first 12 months post‐transplant, (separate) occurrence of ureteral obstruction/stricture during the first 12 months post‐transplant, some other type of urologic complication during the first 12 months post‐transplant, delayed graft function, a UTI (or a recurrent UTI) that required hospitalization during the first 12 months post‐transplant, and graft loss (death‐uncensored) during the first 12 months post‐transplant, respectively. Graft loss was defined as the date of graft failure (return to permanent dialysis, graft nephrectomy, or re‐transplantation) or death, whichever occurred first.

Frequency distributions were determined for baseline and outcome variables that were categorical, and the mean along with standard error (SE) (as well as the median and range of values) were calculated for baseline and outcome variables that were continuous. Tests of association between baseline categorical variables and categorical outcomes were performed using Pearson (uncorrected) chi‐squared tests. Tests of association between baseline categorical variables and continuous outcomes were performed using conventional *t*‐tests. Tests of association between baseline categorical variables and time‐to‐event outcomes were performed using log‐rank tests.

## 3 Results

### 3.1 Patient Demographics

Distributions of selected baseline variables (determined at the time of transplant) are presented in Table 1. Mean recipient age at transplant (±SE) was 11.7 ± 0.5 (range: 2.3–18.9) years; 62.6% (67/107) of recipients were male. Most recipients were either black (non‐Hispanic), 33.6% (36/107), or Hispanic, 44.0% (46/107). The percentage of DD recipients was 66.4% (71/107). Causes of end‐stage renal disease (ESRD) were as follows: acquired in 38.3% (41/107), congenital in 57.0% (61/107), and unknown in 4.7% (5/107). Mean donor age (±SE) was 29.1 ± 1.1 (range: 2–60) years. The percentage of donor kidneys having 1, 2, and 3 donor arteries was 86.0% (92/107), 13.1% (14/107), and 0.9% (1/107), respectively. The percentage of patients that had pretransplant bladder or urinary tract reconstruction was 20.6% (22/107). Of note, all 22 patients had congenital reasons for ESRD, which included renal dysplasia (11 patients), reflux nephropathy (7 patients), prune belly syndrome (5 patients), posterior urethral valves (5 patients), obstructive uropathy (4 patients), and neurogenic bladder (4 patients). Types of pretransplant bladder and/or urinary tract reconstruction included: bilateral native nephrectomy (2 patients), single or bilateral ureteral reimplantation (10 patients), bladder augmentation (6 patients), ureterostomy or vesicostomy (3 patients), and a single native nephrectomy combined with a single ureteral reimplantation (1 patient).

**TABLE 1:** Distributions of selected baseline variables (determined at the time of transplant) (*N* = 107).

Baseline variable	Mean ± SE if continuous; percentage with characteristic if categorical
Recipient age (year)	11.7 ± 0.5 (*N* = 107)
	[Median = 12.9, range: 2.3–18.9]
Recipient age (year)	
< 13	50.5% (54/107)
≥ 13	49.5% (53/107)
Recipient gender	
Female	37.4% (40/107)
Male	62.6% (67/107)
Recipient race/ethnicity	
Black (non‐Hispanic)	33.6% (36/107)
Hispanic	44.0% (46/107)
White (non‐Hispanic)	17.8% (19/107)
Other	5.6% (6/107)^a^
Recipient weight (kg)	38.7 ± 2.1 (*N* = 107)
	[Median = 37.0, range: 11.9–98.5]
Recipient BMI (kg/m^2^)	19.5 ± 0.4 (*N* = 107)
	[Median = 18.4, range: 11.8–36.2]
Re‐transplant status	
Primary	95.3% (102/107)
Re‐transplant	4.7% (5/107)
Preemptive transplant	
No	72.0% (77/107)
Yes	28.0% (30/107)
Pretransplant time on dialysis (months)	18.3 ± 2.0 (*N* = 107)
	[Median = 13.8, range: 0.0–98.9]
Cause of ESRD	
Acquired	38.3% (41/107)
Congenital	57.0% (61/107)
Unknown	4.7% (5/107)
Donor type	
Living	33.6% (36/107)
Deceased	66.4% (71/107)^b^
Number of donor arteries	
1	86.0% (92/107)
2	13.1% (14/107)
3	0.9% (1/107)
Pretransplant bladder/urinary tract reconstruction	
No	79.4% (85/107)
Yes	20.6% (22/107)
Donor age (year)	29.1 ± 1.1 (*N* = 107)
	[Median = 29.0, range: 2.0–60.0]
Donor age (year)	
< 19	18.7% (20/107)
≥ 19	81.3% (87/107)
CIT (h)	15.8 ± 1.2 (*N* = 107)
	[Median = 20.3, range: 0.4–48.6]
CIT (h)	
< 18	43.0% (46/107)
≥ 18	57.0% (61/107)
WIT (min)	30.4 ± 0.5 (*N* = 107)
	[Median = 29.0, range: 18.0–46.0]
KDPI (%)	13.8 ± 1.5 (*N* = 71)
	[Median = 10, range: 1–61]
KDPI (%)	
< 35	95.8% (68/71)
≥ 35	4.2% (3/71)

^a^ Other race/ethnicity included: Indian/Asian (3/107), Middle Eastern (2/107), and Native American (1/107).

^b^ Pediatric en bloc kidneys comprised 1/71 of the deceased donor kidneys.

### 3.2 Urologic Complications

Distributions of selected outcomes variables during the first 12 months post‐transplant are presented in Table 2. Overall, 3.7% of patients (4/107) developed a urologic complication during the first 12 months post‐transplant. Of these, two received a DD kidney, and two received a living donor (LD) kidney. Causes of ESRD in these four patients were: unknown (*N* = 1) and acquired (*N* = 1, FSGS) for the two DD recipients, and congenital (*N* = 2, renal dysplasia) for the two LD recipients. In total, three patients developed a ureteral obstruction/stricture, 2.8% (3/107), with two being due to UPJ obstruction and one being due to the occurrence of multiple kidney stones that occluded the distal ureter/UVJ (thus, initially misdiagnosed as a stricture). Times‐to‐occurrence of these initially diagnosed strictures were 3.9 and 5.5 months post‐transplant for the two UPJ obstructions and 0.6 months post‐transplant for the UVJ occlusion due to kidney stones. No urinary leaks, 0.0% (0/107), were observed during the first 12 months post‐transplant. Additionally, poor bladder compliance along with Grade 4 VUR occurred in one patient at 12 months post‐transplant, 0.9% (1/107). Of note, the patient who experienced a UPJ obstruction at 3.9 months post‐transplant had received a LD kidney that at the time of transplant had a partial ureteral obstruction at the UPJ. The patient who experienced a UPJ obstruction at 5.5 months post‐transplant had developed a ureteral kink (with no stricture) at the UPJ; the reason for its occurrence was unknown. This latter patient was successfully managed with interventional radiology alone (balloon plasty and nephroureteral stent placement). The other three patients, 2.8% (3/107), required surgical intervention: pyeloureterostomy in the patient who developed a UPJ obstruction at 3.9 months post‐transplant (along with balloon plasty and nephroureteral stent placement), ureteral reimplantation in the patient having UVJ obstruction due to multiple kidney stones (in addition to initially receiving balloon plasty and nephroureteral stent placement), and bladder augmentation along with (ureter, ileum, and ileal) Monti Mitrofanoff in the patient having poor bladder compliance.

**TABLE 2:** Distributions of selected outcomes variables during the first 12 months post‐transplant (*N* = 107).

Outcome variable	Mean ± SE if continuous; percentage with characteristic if categorical
Developed DGF	
No	98.1% (105/107)
Yes	1.9% (2/107)
Developed a first urological complication during the first 12 months post‐transplant	
No	96.3% (103/107)
Yes	3.7% (4/107)
Developed a first urinary tract infection requiring hospitalization during the first 12 months post‐transplant	
No	79.4% (85/107)
Yes	20.6% (22/107)
Developed a second urinary tract infection requiring hospitalization during the first 12 months post‐transplant	
No	93.5% (100/107)
Yes	6.5% (7/107)
Developed (death censored) graft failure during the first 12 months post‐transplant	
No	98.1% (105/107)
Yes	1.9% (2/107)
Death with a functioning graft during the first 12 months post‐transplant	
No	100.0% (107/107)
Yes	0.0% (0/107)
Developed (death uncensored) graft loss during the first 12 months post‐transplant	
No	98.1% (105/107)
Yes	1.9% (2/107)
eGFR at 1 month post‐tx (mL/min/1.73 m^2^)^a^	82.6 ± 3.2 (*N* = 106)
	[Median = 78.2, range: 18.9–187.7]
eGFR at 6 months post‐tx (mL/min/1.73 m^2^)^a^	76.4 ± 2.7 (*N* = 106)
	[Median = 76.0, range: 19.7–153.9]
eGFR at 12 months post‐tx (mL/min/1.73 m^2^)^a^	71.4 ± 2.5 (*N* = 105)
	[Median = 70.3, range: 8.6–140.4]

^a^ The race‐based CKD‐EPI formula was used to estimate GFR.

### 3.3 Urinary Tract Infections and Graft Loss

The observed percentage of patients who developed a UTI that required hospitalization during the first 12 months post‐transplant was 20.6% (22/107), with 7/22 and 16/22 of these infections occurring during the first month and 6 months post‐transplant, respectively. In addition, the observed percentage who developed a recurrent UTI requiring hospitalization during the first 12 months post‐transplant was 6.5% (7/107). The observed incidence of UTI requiring hospitalization during the first 12 months post‐transplant was not noticeably higher among the four patients who developed (25.0%; 1/4) versus did not develop (20.4%; 21/103) a urologic complication (N.S.).

The overall percentage of patients who developed graft loss during the first 12 months post‐transplant was 1.9% (2/107); 2 experienced death‐censored graft failure, and 0 experienced death with a functioning graft. One patient experienced graft failure on Day 0 post‐transplant due to renal vein thrombosis (requiring graft nephrectomy); the other patient experienced graft failure at 12 months post‐transplant due to primary disease recurrence (rapidly progressing glomerulonephritis). Neither patient who experienced graft failure during the first 12 months post‐transplant previously developed a urologic complication.

## 4 Discussion

Pediatric kidney transplantation has evolved over decades to become the most effective treatment for end stage kidney disease (ESKD), offering the best long‐term patient survival, improved quality of life, and greater cost‐effectiveness compared to dialysis [10]. Despite new advancements in the transplant surgery [11], the pediatric kidney transplant procedure is more complex than adult kidney transplantation [2], and the risk of developing urologic complications (especially in the early post‐transplant period) remains a significant concern due to its impact on patient morbidity and potentially on graft survival. The incidence of urological complications in the pediatric kidney transplant population has been reported to range from 3% to 34% [1]. To address this issue, the prophylactic use of ureteral stents at the time of kidney transplant has been shown to significantly reduce the incidence of major urological complications such as urinary leakage and ureteral obstruction/stricture compared with no stenting, making it a widely adopted preventive measure [5, 6].

In our study, we report our experience with a novel stentless ureteroneocystostomy technique. The overall incidence of urological complications during the first 12 months post‐transplant in this group was 3.7% (4/107). Excluding the single case of poor bladder compliance/VUR, the percentage of patients who developed either a urinary leak or a ureteral obstruction/stricture was 2.8% (3/107). The use of a ureteroneocystostomy technique without ureteral stent placement at the time of transplant eliminates the uncertainty surrounding optimal timing for stent removal and avoids an additional procedure for stent extraction. UTI represents one of the most common complications following pediatric kidney transplantation, accounting for approximately 37% of cases following LD pediatric kidney transplantation [12], with 28% of patients experiencing recurrent UTIs post‐transplant [13]. Our observed incidence of UTI requiring hospitalization during the first 12 months post‐transplant was acceptably low, 20.6% (22/107). With only 6.5% (7/107) of our cohort developing a recurrent UTI during the first 12 months post‐transplant, these low UTI rates could be related (at least in part) to the absence of routine ureteral stent placement and early Foley catheter removal [8]. An additional advantage in performing stentless ureteral anastomosis was that none of our patients required an extra surgical procedure under anesthesia for stent removal (except for those who required stent placement as treatment for the occurrence of a post‐transplant urologic complication).

An important consideration regarding the routine use of ureteral stent placement at the time of transplant is the incidence of UTIs. In one adult study, the rate of UTI was higher in the stented group, 23.2% compared to 16.6% in the non‐stented group, with a statistically significant difference [14]. In a small pediatric study, the UTI incidences in the non‐stented (*n* = 18) and stented (*n* = 18) kidney transplant groups were similar, 50% versus 39%, respectively [15].

VUR is another complication of the ureteral anastomosis that can occur following kidney transplantation [16], and creation of an anti‐reflux tunnel at the time of the ureteral anastomosis can prevent its occurrence [17]. In our technique, an anti‐reflux tunnel is created by incorporating additional detrusor layers to prevent VUR. VUR is only investigated at our center if the patient becomes symptomatic. It was observed in only one patient, secondary to poor bladder compliance, requiring surgical intervention to resolve the complication.

The routine use of ureteral stent during kidney transplantation has been a topic of considerable interest in the transplant field. This issue has also been the focus of our research, as we have explored the development of a ureteral anastomosis technique that avoids the routine use of a ureteral stent [8].

One study limitation is the fact that the results reported here were not based on a randomized controlled study, nor was there an available control group of transplanted patients at our center who received one of the more conventional extravesical ureteroneocystostomies.

In conclusion, our experience demonstrates that a standardized stentless extravesical ureteroneocystostomy technique in pediatric kidney transplantation can be performed safely with a low incidence of early post‐transplant urologic complications and without an apparent increase in urinary leak or ureteral stenosis. The absence of routine ureteral stent placement may help avoid additional procedures, anesthesia exposure, and stent‐related morbidity while maintaining excellent urological outcomes. These results support selective rather than universal use of ureteral stenting in pediatric kidney transplantation. Larger studies and broader application of this technique by other transplant centers are warranted to further validate these findings.

## Funding

The authors have nothing to report.

## Conflicts of Interest

The authors declare no conflicts of interest.

## Data Availability

The data that support the findings of this study are available from the corresponding author upon reasonable request.
